# Editorial: TB on the rise: but researchers are not sleeping

**DOI:** 10.4314/ahs.v21i3.1

**Published:** 2021-09

**Authors:** James K Tumwine

In this September issue of *African Health Sciences*, we have picked one consistent theme: infections. An interesting infection, and of major concern, is tuberculosis. Our authors have picked several aspects and written for us insightful academic papers, which I will just highlight in passing.

The first TB paper is by Laura Madukaji and colleagues from Nigeria. They report on “Early detection of Pre-XDR TB with line probe assay in a high TB burden country.”^1^ This is followed by a paper from Uganda, in which Andrew Kazibwe and colleagues report on “the yield of different active TB case finding interventions in a large urban TB project, in central Uganda.”^2^. They describe the comparative yield of three active case finding interventions: “health facility-based screening, targeted community screening; and household contact tracing.” They discuss the merits and demerits of the different approaches. Good reading!

Diagnostics continue with an interesting practical paper by Bouzouita, on the “evaluation of PCR *pncA*-restriction fragment length polymorphism and PCR amplification of genomic regions of difference for the identification of *M. bovis* strains in lymph nodes cultures.” 3. They concluded that “PCR *pncA*-RFLP and RD-PCR represent very accurate and rapid tools to identify M. bovis in tuberculosis lymph nodes cultures.” They contend that it can be easily implemented due to “low cost and easy use.”

The other TB papers include a report of a case of “pulmonary cryptococcosis in an immunocompetent child in Uganda” masquerading as TB, which illustrates the importance of the adage: “It is not always Tuberculosis!” 4. This is followed by the twin infection of TB and HIV. Esra Zerdali and others 5 investigate the factors associated with this co-nfection. Work from Ethiopia 6 also discusses TB and HIV co-infection and arrives at fairly similar conclusions: that marital status, education level, CD4 cell count and clinical stage of the HIV were associated with HIV/TB coinfection.

Clearly, TB is still a serious illness in our region. There is need for more work in this area, especially its interaction with emergency infections such as Covid-19, Ebola and malaria. The ball is in the court of our budding and established scientists!
